# Response to Wang and Hunter: A Systematic Review and Meta-Analysis of the Association between Self-Reported Diarrheal Disease and Distance from Home to Water Source

**DOI:** 10.4269/ajtmh.2011.10-0591a

**Published:** 2011-03-04

**Authors:** Saleena Subaiya, Sandy Cairncross

**Affiliations:** London School of Hygiene and Tropical Medicine, Keppel Street; London WC1E 7HT, United Kingdom; E-mails: saleena.subaiya@gmail.com; sandy.cairncross@lshtm.ac.uk

Dear Sir:

In response to the review of Wang and Hunter of the association between self-reported diarrheal disease and distance from home to water source,^1^ we support their choice of distance to water source as a risk factor to study. It has been neglected lately, in the enthusiasm for studies of water quality.^2^

A number of studies of time to water source (TTWS) and quantity of water used at the household level have found that when round trip TTWS is within 30 minutes, which is equivalent to 1 km each way if the average person walks at 4 km/hour, water use is relatively inelastic. However, for sources greater than 30 minutes or 1 km away, water consumption decreases.^3^–^5^ The studies included in this review involve comparison points much less than 30 minutes or 1 km, which may be insufficient to cause a true difference in diarrheal morbidity. One study that was not included in this review compared groups that have to walk farther than 1,000 meters and less than 100 meters to their water source, and found that diarrheal risk increased with distance to source (unadjusted odds ratio = 3.80, 95% confidence interval = 1.89–4.21).^6^

The pooled estimate calculated in the review of Wang and Hunter's was drawn predominantly from univariate analyses. One of their included studies^7^ (as well as two not included^8^,^9^) concluded from multivariate analysis adjusting for socioeconomic status, age, and maternal education that evidence for an association between TTWS and diarrheal morbidity does not exist.^7^–^9^

Although this review highlights the importance of the TTWS indicator, there is insufficient evidence for an association between distance to water source and diarrheal illness at this time. This evidence is the result of mixed findings of studies, the existence of numerous methodologic flaws in design, and the risk of bias. However, the largest study performed on this subject with the least methodologic flaws demonstrated a positive score test for linear trend with increasing distance to water source and diarrheal morbidity,^10^ thus supporting the conclusion of Wang and Hunter that more well-designed studies are needed in this area.
